# The Carbon Emission Reduction Effect of Tourism Economy and Its Formation Mechanism: An Empirical Study of China’s 92 Tourism-Dependent Cities

**DOI:** 10.3390/ijerph19031824

**Published:** 2022-02-05

**Authors:** Yun Tong, Rui Zhang, Biao He

**Affiliations:** 1 School of Tourism, Hainan University, Haikou 570228, China; tongyuntour@163.com (Y.T.); hebiaotaylor@163.com (B.H.); 2 School of Business, Hubei University, Wuhan 430062, China; 3 Hainan Province Holistic Tourism Research Base, Haikou 570228, China; 4 Academician Desheng Wu Station of Hainan Province, Haikou 570228, China

**Keywords:** tourism economy, carbon emission intensity, multiple pathways, structural equation model

## Abstract

The tourism economy is regarded as an effective way to realize regional sustainable development. Hence, it is of great significance to explore whether and how tourism economy can alleviate regional carbon emission intensity. To this end, a structural equation model (SEM) reflecting the multiple pathways of the carbon emission reduction effect of tourism economy was constructed based on 92 tourism-dependent cities in China, and the existence and formation mechanism of the carbon emission reduction effect of tourism economy were empirically tested. The main findings are as follows: (1) The tourism economy has a significant carbon emission reduction effect in China. Although the direct impact of tourism economy on carbon emission intensity is significantly positive, the indirect impact is significantly negative and stronger than the direct impact. (2) The carbon emission reduction effect of tourism economy presents multiple pathways characteristics. There are single intermediary pathways such as Tourism Economy → Environmental Regulation → Carbon Emission Intensity, Tourism Economy → Opening-Up → Carbon Emission Intensity, and dual intermediary pathways such as Tourism Economy → Opening-Up → Industrial Development → Carbon Emission Intensity, Tourism Economy → Opening-Up → Innovation Capacity → Carbon Emission Intensity. (3) The formation mechanism of the carbon emission reduction effect of tourism economy presents obvious spatial heterogeneity.

## 1. Introduction

Global climate change is a major challenge facing all countries, which profoundly affects the survival and development of humankind [1,2]. In the fourth global climate assessment, the Intergovernmental Panel on Climate Change (IPCC) pointed out that human activities and greenhouse gas emissions are the main causes of global warming [3]. Therefore, reduction in greenhouse gas emissions has become an important mission for all humankind to cope with the challenge of global warming, and countries around the world have dedicated numerous efforts to achieving this [4,5].

Since the implementation of the reform and opening-up more than 40 years ago, China has seen miraculous economic growth [6], and its share of the global GDP rose from 1.75 percent in 1978 to 16.04 percent in 2018 [7]. However, this unprecedented economic boom has come at the cost of relatively serious environmental pollution problems. According to the research report *Challenges of Maintaining Global Warming Below 2 °C* by the Tyndall Center for Climate Change Research, China, as the world’s largest energy consumer, ranks first in the world in terms of total carbon emissions [8], and its carbon emissions amounted to 37.2 billion tons in 2018 [9]. The Chinese government has realized that China should participate extensively in international climate cooperation and undertake its corresponding international obligations [10,11]. Hence, at the 75th United Nations General Assembly (UNGA), the Chinese government proposed China’s commitment and strategic goals to reach peak carbon emissions by 2030 and achieve carbon neutrality by 2060 [12].

**Figure 1:**
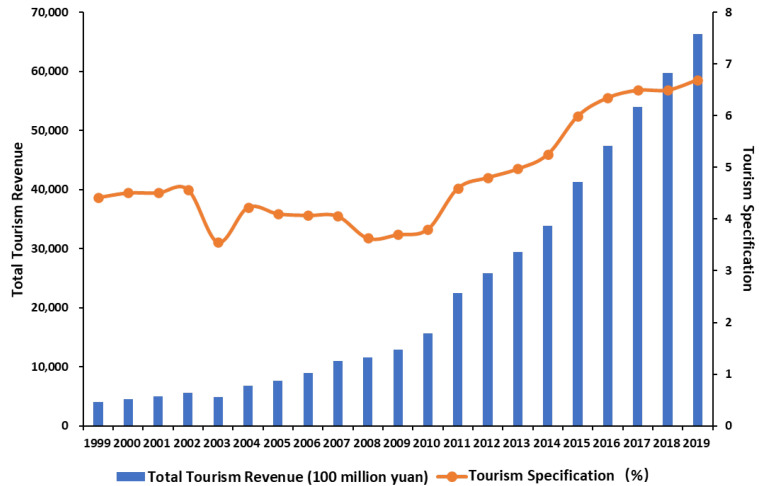
Total tourism revenue and tourism specialization in China (1999–2019).

Industrial structure is an important factor affecting the level of carbon emission [13,14]. Therefore, building a green and low-carbon economic system is an important way for China to achieve the goals of carbon peak and carbon neutrality. It is also conducive to China’s realization of the United Nations Sustainable Development Goals (SDGs) to ensure sustainable consumption and production patterns. Many international organizations, such as the United Nations (UN), the International Monetary Fund (IMF), and the World Bank (WB), believe that the tourism industry is a key economic sector for promoting the transformation of the economic system from “brown” to “green” [15]. Can tourism economy effectively contribute to the achievement of carbon peak and carbon neutrality goals and become a reliable segment of the green and low-carbon economic system in China? In practical terms, the tourism industry has been considered by the Chinese government as a strategic pillar industry of the national economy in recent years due to the improvement of national living standards and the increase in tourism demand. According to statistics from the Ministry of Culture and Tourism of the People’s Republic of China, the scale of China’s tourism industry continues to expand (Figure 1). Indeed, its comprehensive contribution to national economic growth and employment has exceeded 10% [16].

Theoretically, based on panel data or time-series data analysis, some studies have indicated that tourism economy could significantly reduce the regional carbon emission intensity (i.e., carbon emissions per unit GDP; see Section 4.2.2 for more details) of destinations [17,18], although it has generated a certain amount of carbon emissions [19]. In this context, we aim to answer the following three questions: (1) Can the development of tourism slow down the overall carbon emission intensity of the destination? (2) If the tourism economy has a carbon emission reduction effect, what is the mechanism for tourism to reduce carbon emission intensity? It is worth mentioning that the existing literature lacks research on this issue. (3) Furthermore, does the formation mechanism have spatial heterogeneity?

To answer the above questions, we adopted the panel data of 92 tourism-dependent cities in China from 2005 to 2016 to construct a structural equation model (SEM) and empirically tested the existence and formation mechanism of the carbon emission reduction effect of tourism economy. In other words, we separately explored the direct effects of tourism economy on carbon emission intensity and the indirect effects of tourism economy on carbon emission intensity through industrial development, environmental regulation, and opening-up. In addition, given that tourism-dependent cities are distributed among various regions in China, the spatial heterogeneity of the pathway characteristics of tourism economy affecting carbon emission intensity is further discussed. The potential contributions of this research are as follows: (1) This research provides an empirical framework combining tourism economy and carbon emission intensity to deeply understand the environmental externalities of the tourism industry. In technical terms, we applied SEM to investigate the multiple pathways of tourism economy’s influence on carbon emission intensity. Namely, the formation mechanism of the carbon emission reduction effect of tourism economy has been verified. (2) Existing research on the interaction between tourism and carbon emission was mostly based on the national and provincial levels. To this end, we utilized China’s tourism-dependent cities as a research sample to supplement empirical research on a prefecture-city level. (3) From a practical point of view, this research is conducive to building a policy system for the tourism industry to promote green and low-carbon transition and is of great value for tourism-dependent cities to explore green and low-carbon transition pathways.

The remainder of this study proceeds as follows: Section 2 provides a literature review. Section 3 presents the theoretical analysis and research hypothesis. Section 4 introduces the methods and data sources. Section 5 reports and analyzes the empirical results based on SEM. Section 6 gives concluding remarks.

## 2. Literature Review

Tourism development, such as the construction of tourist attractions, the development of accommodation and catering facilities, and tourism activities, have extensive impacts on the environment and ecology of destinations [20]. Existing studies have shown that tourism can have adverse effects on the environment. For example, several scholars have revealed that while tourism actively promotes the development of the economy, it also causes adverse effects in terms of water pollution [21,22]. In addition, several studies have found that negative impacts on the environment and biodiversity are most pronounced near tourist resorts [23,24]. Some scholars have found that tourism seems to be a major contributor to greenhouse gas emissions, and its air pollution cannot be ignored [25,26]. Among the above issues, the impact on greenhouse gas emissions is an important topic of long-term academic attention.

According to estimates by the World Tourism Organization (UNWTO) (though the estimate does not include carbon emissions from food, accommodation, and shopping, so the actual value may be higher than the estimated result), tourism carbon emissions account for 4.9% of global anthropogenic carbon emissions [27]. Tourism is one of the sources of global greenhouse gas emissions. In other words, the total amount of carbon emissions generated by tourism is hard to ignore, but tourism economy has a comprehensive impact on the destination economy system, environment system, and social system [28,29], which may have a mitigating effect on regional carbon emission intensity.

Based on our understanding of tourism’s carbon emissions [30,31], the academic community has carried out extensive discussions on the effects of tourism economy on regional carbon emissions. Most of the literature has confirmed that there is a significant causal relationship between tourism economy and regional carbon emissions.

On one hand, some studies supported the view that tourism economy has a carbon emission reduction effect. Lee et al. found that the tourism economy of EU countries not only can promote domestic economic growth but also has a long-term co-integration relationship with carbon emissions, which could significantly curb carbon emissions [32]. Dogan et al. used a heterogeneous panel estimation with cross-sectional dependence to study the relationship among carbon emissions, real income, energy consumption, and tourism economy in EU countries and reached the same conclusion [33]. Paramati et al. conducted panel cointegration and long-term elasticity tests on the annual data of EU countries and found that there was a long-term equilibrium relationship between tourism investment and carbon emission, and tourism investment significantly reduced carbon emission [34]. Alam et al. investigated the dynamic role of tourism investment on tourism development and carbon emissions and found that long-term elasticity and short-term causality, concluding that tourism investment in 10 tourism-dependent countries around the world also inhibited carbon emissions [35]. In addition, Brahmasrene et al. utilized a cointegration test and panel regression model to conduct empirical research on 10 Southeast Asian countries, and they came to the same conclusion that tourism economic growth was conducive to destination carbon emission reduction [36]. Lee et al. studied the impact of the tourism and hotel industries in Korea on economic growth and carbon emissions and found that the growth of the tourism economy in Korea could also reduce carbon emissions [37].

In contrast, some studies determined that tourism economy could significantly aggravate destination carbon emissions. Rabindra et al. concluded that for every 1% increase in tourist arrivals in Nepal, carbon emissions increased by 0.98%, which reminded the government to promote the green tourism agenda [38]. Len et al. found that the tourism economies of both developed and developing economies had a significant role in promoting carbon emissions, but the effect in developed countries was even more pronounced [39]. Similarly, Balli et al. concluded that tourism statistically had a significant impact on the economic growth of Mediterranean countries, leading to increases in regional carbon emissions [40]. Danish et al. found that tourism could not only significantly promote the economic growth of BRICS countries but also increase regional carbon emissions and reduce environmental quality [41]. Zhang et al. utilized the VECM Granger causality method for the first time to study short-term and long-term causality among tourism, economic growth, energy consumption, and carbon emissions in 30 provinces in China, and they indicated that tourism could promote economic growth and increase regional carbon emissions [42].

In addition, the nonlinear relationship between tourism economy and regional carbon emissions was also explored based on the Environmental Kuznets Curve (EKC) framework. Paramati et al. revealed the nonlinear impact of tourism on carbon emissions using 26 developed economies and 18 developing economies as the research sample. Specifically, due to differences in national income, the impact of tourism in developed economies on carbon emissions was smaller than that in developing economies [43]. This provided evidence for the EKC hypothesis between tourism economy and carbon emissions. Wang et al. found that with increases in GDP per capita, there were significant U-shape and inverted U-shape relationships among China’s tourism reception, tourism consumption per capita, and regional carbon emissions per capita [44].

However, the above studies still have certain limitations, including the following research gaps. (1) Due to differences in research samples, proxy indicators, and research methods, the effects of tourism economy on regional carbon emissions have been not yet conclusive. (2) Existing studies did not provide further explanations for the relationship between tourism economy and regional carbon emissions but only tried to explore the interactive relationship of them. (3) In addition, the existing literature mostly focused on research based on the national level and lacked detailed discoveries on the prefecture-city level. Given this, we adopted 92 tourism-dependent cities in China as a research sample and not only estimated the effect of tourism economy on regional carbon emissions but also utilized the SEM method to reveal the multiple pathways of the carbon emission reduction effect of tourism economy.

## 3. Theoretical Analysis and Research Hypothesis

### 3.1. The Direct Impact of Tourism Economy on Carbon Emission Intensity

The direct impact refers to the direct influence of tourism economy on carbon emission intensity without intermediate variables, and it is equal to the path coefficient of tourism economy to carbon emission intensity.

Following pioneering work to estimate global carbon emissions from tourism, Gössling found that tourism has a typically high carbon attribute, and its carbon consumption intensity is higher than that of daily life [45,46]. First, tourism transportation, tourism accommodation, tourism activities, and many other segments have been producing carbon emissions [47]. In particular, the large-scale spatial tourism flow boosted by modern transportation such as aviation and cruise ships, the high energy consumption of the star-rated accommodation industry, and the major investment projects in tourism have been aggravating the greenhouse gas emissions of tourism economy itself. Public service facilities and infrastructure construction that tourism development relies on also lead to a large amount of carbon emissions. Second, the carbon emission efficiency of tourism is often lower than that of advanced manufacturing, high-tech industries and tertiary industries such as education and finance. Finally, the impact of tourism economy on carbon emission intensity may be further amplified because tourism economy accounts for a high proportion of the national economy in tourism-dependent cities.

On this basis, this study proposes the theoretical Hypothesis 1.

**Hypothesis 1** **(H1):** *Tourism economy can directly increase the regional carbon emission intensity of tourism-dependent cities*.

### 3.2. The Indirect Impact of Tourism Economy on Carbon Emission Intensity

Indirect impact refers to the effects of the tourism economy on carbon emission intensity by affecting one or more intermediate variables, and the value of indirect impact is the sum of the products of path coefficients.

#### 3.2.1. Single Mediation Pathway

(1) Tourism Economy → Environmental Regulation → Carbon Emission Intensity

This pathway is to explore the impact of tourism economic and environmental regulations on carbon emission intensity. Tourism economy can prompt the government to implement more stringent environmental regulations to ensure that the environmental system can support sustainable tourism development, since it is a necessary condition for tourism development to maintain a good ecological environment [48]. The intensity of environmental regulation can restrain carbon emission intensity [49,50]. Accordingly, theoretical Hypothesis 2 is proposed.

**Hypothesis 2** **(H2):** *Tourism economy can reduce regional carbon emission intensity by strengthening environmental regulation*.

(2) Tourism Economy → Industrial Development → Carbon Emission Intensity

This pathway is to explore the impact of the deindustrialization effect of tourism economy on carbon emission intensity. Existing studies concluded that tourism economy had a deindustrialization effect [51]. On one hand, the large amount of land, labor, and other production factors linked to tourism development can cause the price of these factors to rise, which can squeeze the space for industrial development. On the other hand, tourism which relies on high-quality tourism resource endowment can bring economic prosperity in the short term and transfer labor force, capital, and other elements to it [52]. Industrial development usually has the characteristics of high energy consumption and high emissions, which can improve carbon emission intensity. Therefore, theoretical Hypothesis 3 is proposed.

**Hypothesis 3** **(H3):** *Tourism economy can reduce regional carbon emission intensity by inhibiting industrial development*.

(3) Tourism Economy → Opening-Up → Carbon Emission Intensity

This pathway is to explore the impact of the opening-up effect of tourism economy on carbon emission intensity. First, tourism development can improve public services and infrastructure, optimize traffic location conditions, expand the visibility of destinations, and further expand the opening-up. What is more, as an important part of the tourism industry, business tourism and MICE tourism can enable potential investors to obtain first-hand information about the destination and bring more investment opportunities to the destination, thus promoting its opening-up. Second, we use the capital stock of foreign direct investment (FDI) per capita to represent the opening-up. FDI’s impact on the carbon emission intensity of the destination cannot be judged, since it may have a pollution haven effect or pollution halo effect [53]. Hence, this study further puts forward theoretical Hypothesis 4.

**Hypothesis 4** **(H4):** *Tourism economy can significantly affect regional carbon emission intensity by promoting opening up*.

#### 3.2.2. Dual Mediation Pathway

(1) Tourism Economy → Environmental Regulation → Industrial Development → Carbon Emission Intensity

The increase in the intensity of environmental regulations can reduce the space for industrial development through the environmental governance of industrial enterprises. Then, industrial development may promote regional carbon emission intensity. Therefore, theoretical Hypothesis 5 is proposed.

**Hypothesis 5** **(H5):** *Tourism economy can restrain industrial development by strengthening environmental regulations and then reduce regional carbon emission intensity*.

(2) Tourism Economy → Opening-Up → Industrial Development → Carbon Emission Intensity

The tourism industry, as an open economy, can promote the opening-up of destinations and attract FDI. Affected by the foreign investment admission list policy, FDI focuses on manufacturing as the main investment area. Therefore, FDI may affect carbon emission intensity indirectly by influencing industrial development. Accordingly, theoretical Hypothesis 6 is proposed.

**Hypothesis 6** **(H6):** *Tourism economy can promote industrial development by improving opening-up and then aggravate destination carbon emission intensity*.

(3) Tourism Economy → Opening-Up → Innovation Capacity → Carbon Emission Intensity

If the opening-up effect of tourism economy exists, the advanced technology introduced through FDI can help cities to improve their innovation capabilities. Therefore, FDI may affect carbon intensity by influencing innovation capacity indirectly. On this basis, this study further puts forward theoretical Hypothesis 7.

**Hypothesis 7** **(H7):** *Tourism economy can enhance innovation capacity by improving the level of opening-up and then reduce destination carbon emission intensity*.

**Figure 2:**
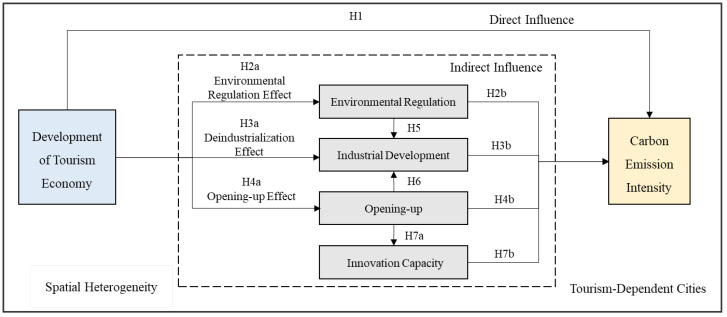
Mechanism of the influence of tourism economy on regional carbon emission intensity.

Based on the above analysis, the mechanism of the influence of the tourism economy on regional carbon emission intensity is constructed as the theoretical framework of this empirical study, as shown in Figure 2.

## 4. Method and Data Sources

### 4.1. Structural Equation Model (SEM)

The tourism economy has multidimensional effects on the economy, environment, society, and policy of destinations, all of which have been widely discussed [54,55]. Therefore, conventional econometric analysis methods such as the mediation effect model cannot effectively reveal the systematic and multichannel characteristics of tourism economy in reducing carbon emission intensity.

To empirically examine the theoretical framework of Figure 2, SEM is utilized. It could effectively identify the formation mechanism of the carbon emission reduction effect of tourism economy. SEM is widely applied in the fields of economics, management, and psychology [56]. It is a multivariate data analysis tool that effectively explores the interrelationship and complex relationships among multiple variables and which can accurately measure the pathway coefficient of each variable and test the relationship among latent variables (variables that cannot be directly observed and need to be measured using observable variables [57,58]), observation variables, and error variables in the model. Furthermore, the direct effect, indirect effect, and total effect of exogenous variables on endogenous variables can be obtained. A typical SEM revealing the exogenous and endogenous variables can be represented as shown on Equation (1) [59].

(1) η=Bη+Γξ+ζ

where *η* represents the vector of endogenous variables, *ξ* denotes the vector of exogenous variables, *B* represents the matrix of coefficients of the endogenous variables, *Γ* is the matrix of coefficients of the exogenous variable, and *ζ* represents the temporarily unexplainable part of the equation, namely, the residual of the structural equation.

### 4.2. Specification of Variables and Data Sources

#### 4.2.1. Research Sample

**Figure 3:**
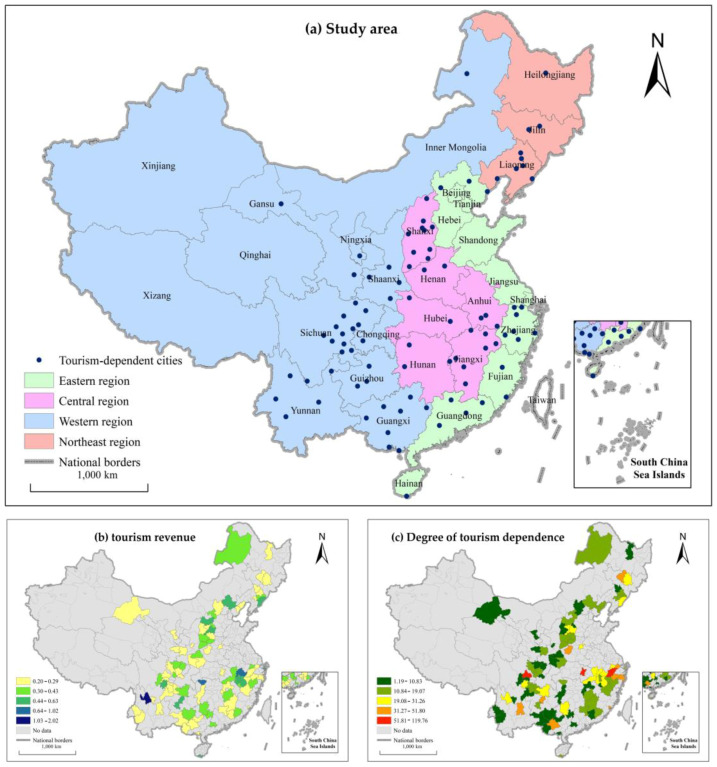
Location of the research sample.

The research sample comprises 92 tourism-dependent cities in China. Referring to Holzner et al. [60], we used the tourism industry dependence index (i.e., the proportion of total tourism revenue in GDP) as a measurement standard and selected cities with an average index of more than 20% in the past three years during the study period (2005–2016). Then, a panel data set of tourism-dependent cities was constructed. The spatial distribution of tourism-dependent cities is shown in Figure 3a. The tourism revenue and degree of tourism dependence of the research sample are shown in Figure 3b,c, respectively.

#### 4.2.2. Variables of the Structural Equation Model

According to existing research and data availability, the definition of each variable of the model and its calculation method are shown in Table 1. To eliminate heteroscedasticity, we utilized the logarithm to process the original data uniformly. The descriptive statistics and data sources of variables are shown in Table 2.

(1) Carbon emission intensity (ln*CI*) was measured by carbon emissions per unit GDP whose basic indicators are night light data and GDP [61,62]. The total carbon emission of each city was calculated by summing up the carbon emission data of China’s county-level administrative regions from Chen et al. [63]. They solved the discontinuity problem of DMSP/OLS and NPP/VIIRS satellite images based on a particle swarm optimization-back propagation (PSO-BP) algorithm and obtained stable and continuous night light data. Further, carbon emission data were be calculated by fitting the relationship between night light and carbon emission. According to Chen et al., the R^2^ of the carbon emission data obtained by inversion of night light data and energy accounting is as high as 0.998, which indicates that night light data are suitable for measuring carbon emissions.

(2) Tourism economy (ln*tour*) was measured by the proportion of total tourism revenue in GDP [64], reflecting the importance of the tourism economy in the economic system. Total tourism revenue is the sum of domestic tourism revenue and inbound tourism revenue (converted into RMB at the annual average exchange rate). The total tourism revenue and GDP were obtained from the China City Statistical Yearbook (2006–2017).

(3) Industrial development (ln*ind*) is a key factor affecting carbon emissions [65]. The high energy consumption characteristics of industrial development can have a significant impact on carbon emissions [69]. In addition, Chao et al. found that the expansion of tourism would lead to deindustrialization [70]. Hence, the industrial development is incorporated into SEM. Referring to Dong et al., we used the proportion of industrial added value in GDP to measure industrial development [65]. The industrial added value was obtained from the China City Statistical Yearbook (2006–2017).

(4) Innovation capacity (ln*innov*) is an important driving force for solving the contradiction between economic development and environmental protection [66]. He et al. concluded that environmental quality could be improved by improving innovation capabilities [71]. Therefore, innovation capacity was incorporated into the construction of SEM. Referring to the ideas of Kou et al. [72], innovation capacity was measured using the innovation index of prefecture-level cities in *The Report on Chinese City and Industrial Innovation* released by the Industrial Development Research Center of Fudan University. The report estimates the value of invention patents using the patent renewal model proposed by Pakes and Schankerman [73], then sums the value of each patent in the cities to obtain the innovation index of prefecture-level cities. The original data source is the invention patents applied for in the State Intellectual Property Office of China, which covers all economic sectors.

(5) Opening up (ln*open*) has a certain time lag. Inspired by Chishti et al. [67], we measured opening-up via the capital stock of FDI per capita. The stock of FDI was calculated using the perpetual inventory method, and the data of FDI were obtained from the China City Statistical Yearbook (2006–2017). Based on a timescale from 2005 to 2016, the stock of city *i*’s FDI in 2005 was:

(2) $${\mathrm{FDI}}_{2005}^{i}=fdi_{2005}^{i}/\left(g^{i}+\alpha \right)$$

where $fdi_{2005}^{i}$ is the FDI of city *i* in 2005, calculated at the exchange rate of the year; $g^{i}$ denotes the growth rate of GDP per capita of city *i* from 2005 to 2016; and the annual depreciation rate was set at 6%. Concerning the formula of the perpetual inventory method, the calculation method of FDI stock in subsequent years from 2005 onwards is as presented in Equation (3):

(3) $${\mathrm{FDI}}_{i}\left(t\right)={\mathrm{FDI}}_{i}\left(t-1\right)-\alpha {\mathrm{FDI}}_{i}\left(t-1\right)+fdi_{i}\left(t\right)$$

(6) Environmental regulation can be examined objectively from the perspective of regulatory results [68]. Therefore, the construction of the environmental regulation index (ln*envi*) draws on the ideas of Li et al. [74]. Specifically, we used the industrial wastewater discharge per unit output value and the industrial SO_2_ emissions per unit output value based on the availability of data to calculate the environmental regulation index. The lower the index, the greater the pollution discharge and the weaker the intensity of environmental regulation. The data of industrial wastewater discharge and industrial SO_2_ discharge were obtained from the China City Statistical Yearbook (2006–2017). The calculation steps are as follows.

The first step is to perform extremely poor standardization treatment on the industrial wastewater discharge per unit output value and the industrial SO_2_ discharge per unit output value of each city, as shown in Equation (4).

(4) $$UE_{ij}^{s}=\left[UE_{ij}-min\left(UE_{j}\right)]/\;[max\left(UE_{j}\right)-min\left(UE_{j}\right)\right]$$

where $UE_{ij}^{s}$ represents the normalized result, $UE_{ij}$ denotes the emission amount of pollutant *j* per unit output value of city *I*, and $max\left(UE_{j}\right)$ and $min\left(UE_{j}\right)$ respectively represent the maximum and minimum emissions per unit output value of pollutant *j* in the research sample.

The second step is to set the weight of industrial wastewater discharge per unit output value of each city as α_1_ and the weight of industrial SO_2_ emissions per unit output value as α_2_. For the convenience of calculation, we assumed that α_1_ = α_2_ = 0.5.

The third step is to calculate the comprehensive index of environmental regulation of city *i*, as shown in Equation (5).

(5) $$EVNI_{i}=\frac{1}{\frac{1}{2}\sum_{j=1}^{2}\alpha_{j}UE_{ij}^{s}}$$

## 5. Empirical Results and Discussion

### 5.1. Temporal and Spatial Characteristics of Carbon Emissions in Tourism-Dependent Cities

**Figure 4:**
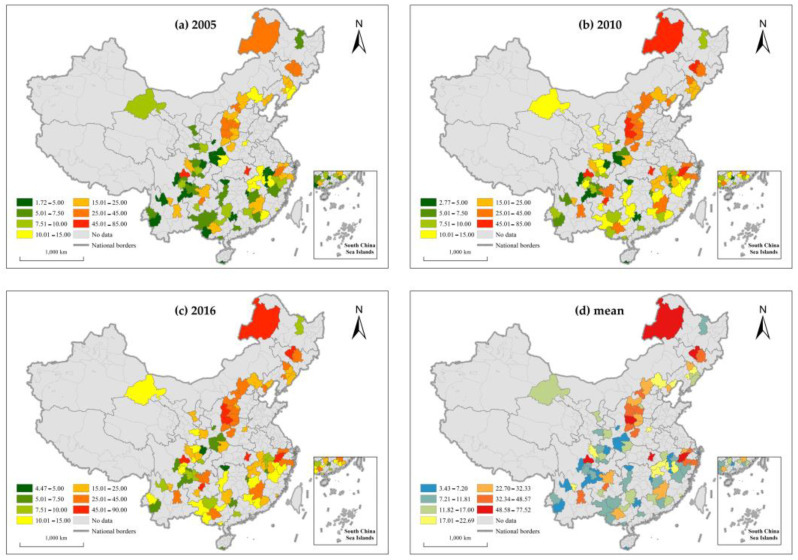
Temporal and spatial patterns of total carbon emissions in tourism-dependent cities.

**Figure 5:**
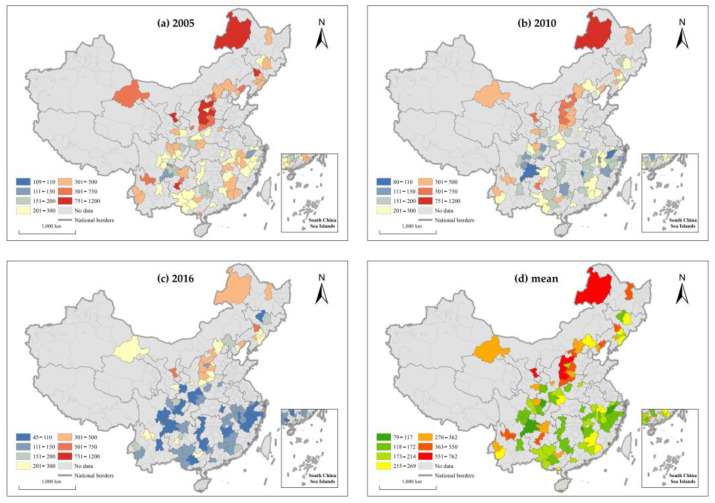
Temporal and spatial patterns of carbon emission intensity in tourism-dependent cities.

To reveal the evolutionary characteristics, the ArcGIS was applied to visualize the total carbon emission (Figure 4a–c) and carbon emission intensity (Figure 5a–c) of tourism-dependent cities in 2005, 2010, and 2016. The Jenks natural breaking point method was used to display the spatial pattern the total carbon emissions (Figure 4d) and carbon emission intensity (Figure 5d) of tourism-dependent cities during the entire study period.

The results show that (1) in terms of time, the carbon emission intensity of tourism-dependent cities shows an overall downward trend during the study period, which indicates that the efficiency of carbon emission has improved. Given the overall upward trend of carbon emissions during the study period, the reduction in carbon emission intensity is mainly attributed to the substantial growth of economic output of tourism-dependent cities. (2) It can be seen from the spatial pattern that both total carbon emission and the carbon emission intensity show obvious north–south spatial differentiation. The total carbon emission of tourism-dependent cities in the north is higher than that in the south, while carbon emission intensity in the south is lower than that in the north. The reason is that the northern region has mainly depended on industry as the main driving force to promote the development of urbanization, so its total carbon emissions and carbon emission intensity are higher. It is worth mentioning that both total carbon emission and carbon emission intensity form a high-value cluster in the Shanxi province, which is consistent with the study of Song et al. [75].

### 5.2. The Formation Mechanism of the Carbon Emission Reduction Effect in Tourism Economy

**Figure 6:**
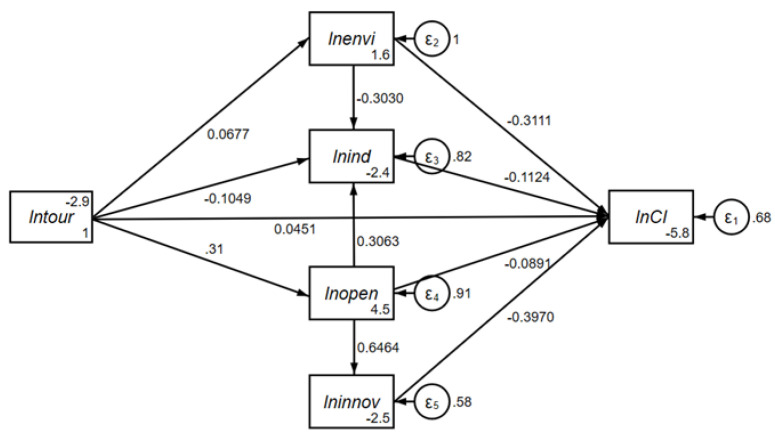
The estimation results of SEM.

Taking 92 tourism-dependent cities in China as the research samples, the maximum likelihood estimation method in STATA software (StataCorp, College Station, TX, USA) was utilized to estimate the parameters of SEM (Figure 2), and the estimation results, including standardized estimation coefficient, standard error, Z-value, and *p*-value, are reported (see Figure 6 and Table 3 for more detail). In terms of the overall fitness of the model, the comparative fit index (CFI) is 0.942 greater than 0.9, and the standardized root mean squared residual (SRMR) is 0.068 lower than 0.08, which indicate that the empirical model is acceptable.

#### 5.2.1. The Direct Impacts of Tourism Economy on Carbon Emissions

In terms of the direct effect of tourism economy (ln*tour*) on carbon emission intensity (ln*CI*), an increase in ln*tour* by 1% in tourism-dependent cities would significantly increase ln*CI* by 0.045%. Therefore, theoretical hypothesis H1 is established, which suggests that the increase in tourism specialization has intensified carbon intensity. In other words, the overall carbon emission efficiency of the destination is reduced. Not only do tourism transportation, tourism accommodation, tourism activities, and other segments emit a large amount of carbon dioxide, the development of China’s tourism at this stage also has project-driven characteristics. Traditional hotels, scenic spots, and tourist infrastructure at an early stage of construction also produce massive carbon emissions, and economic output is often unable to balance carbon emissions in the short term. 

#### 5.2.2. The Single Intermediary Pathway of Tourism Economy to Carbon Emission

There are multiple pathways for the influence of tourism economy on regional carbon emission intensity. 

First, the pathway coefficient of ln*tour* on environmental regulation (ln*envi*), industry development (ln*ind*), and opening-up (ln*open*) was 0.0677, 0.1049, and 0.660, respectively, indicating that tourism economy has a significant environmental regulation effect, deindustrialization effect, and opening-up effect, which reflect the comprehensive impact of tourism economy on destinations.

Second, environmental regulation can significantly reduce carbon emission intensity, that is, there is a pathway of ln*tour* → ln*envi* → ln*CI*. Theoretical hypothesis H2 is thus valid. The coefficient of innovation capacity (ln*innov*) on ln*CI* was −0.0891, namely, there is a pathway of ln*tour* → ln*open* → ln*CI*, and theoretical hypothesis H4 is thus established.

Third, the estimation results of SEM also show that industrial development can reduce carbon emission intensity, which is similar to the research of Yu et al. [76]. Just as in the previous analysis, tourism economy directly increases carbon emission intensity, which indicates that tourism economy has higher carbon emission intensity. In the context of tourism-dependent cities, industrial sectors (especially advanced manufacturing and high-tech enterprises) have higher carbon emission efficiency than tourism economy. Therefore, industrial development has restrained the carbon emission intensity of tourism-dependent cities. Based on the above analysis, tourism economy cannot produce regional carbon emission reduction effects via the deindustrialization effect, which means that theoretical hypothesis H3 is not established.

#### 5.2.3. The Dual Intermediary Pathway of Tourism Economy to Carbon Emission

Further, the dual intermediary pathway of tourism economy to carbon emission intensity was investigated. The results are as follows: (1) Because of the inhibitory effect of industrial development on carbon emission intensity, there is no dual intermediary pathway of ln*tour* → ln*envi* → ln*ind* → ln*CI*. Theoretical hypothesis H5 is thus not established. (2) Due to the increase in opening up, industrial development and innovation capacity are significantly promoted. Meanwhile, the pathway coefficient of ln*innov* on ln*CI* is −0.3970. Therefore, tourism economy can promote a reduction in carbon emission intensity by expanding the opening-up effect. Additionally, the two dual intermediary pathways of ln*tour* → ln*open* → ln*ind* → ln*CI* and ln*tour* → ln*open* → ln*innov* → ln*CI* can be formed. Therefore, theoretical hypotheses H6 and H7 are valid.

#### 5.2.4. Further Analysis Based on Effect Decomposition

The above analysis preliminarily reveals the multiple pathways of tourism economy affecting carbon emission intensity. Due to the complexity of single intermediary and double intermediary, it is necessary to decompose the direct effect, indirect effect, and total effect. The results are summarized in Table 4.

(1) The total effect of the ln*tour* on ln*CI* is −0.0544 at the 1% significance level, which indicates that the improvement of tourism specialization in tourism-dependent cities is conducive to a reduction in carbon emission intensity. However, if we only examine the direct impact of tourism economy on carbon emissions, its direct effect on ln*CI* is 0.0313 at the 10% significance level. The indirect effect formed by multiple pathways is −0.0857 at the 1% significance level, which reverses the direct adverse impact of tourism economy on carbon emission intensity. The above empirical evidence shows that the carbon emission reduction effect of tourism economy mainly depends on the indirect restraint pathway rather than a direct impact. It also suggests that the development of the tourism industry in China is not green and low-carbon in practical terms, and environmental output, such as greenhouse gas emissions, should not be ignored. In addition, the ecological efficiency of tourism needs to be improved.

(2) The effect decomposition results also show other important pathways that affect the carbon emission intensity of tourism-dependent cities. First, opening up has significant negative effects on carbon emission intensity, both direct and indirect, of which the direct effect accounts for about 23.4% of the total effect. This shows a significant pollution halo effect. Second, the inhibitory effect of environmental regulation on carbon emission intensity is mainly reflected in the direct effect. The enhancement of environmental regulation inhibits industrial development, but the total effect of ln*ind* on ln*CI* is −0.0989 at the 1% significance level. Hence, the indirect effect of environmental regulation on carbon emission intensity is positive.

### 5.3. Spatial Heterogeneity of the Formation Mechanism of Carbon Emission Reduction Effect in Tourism Economy

**Figure 7:**
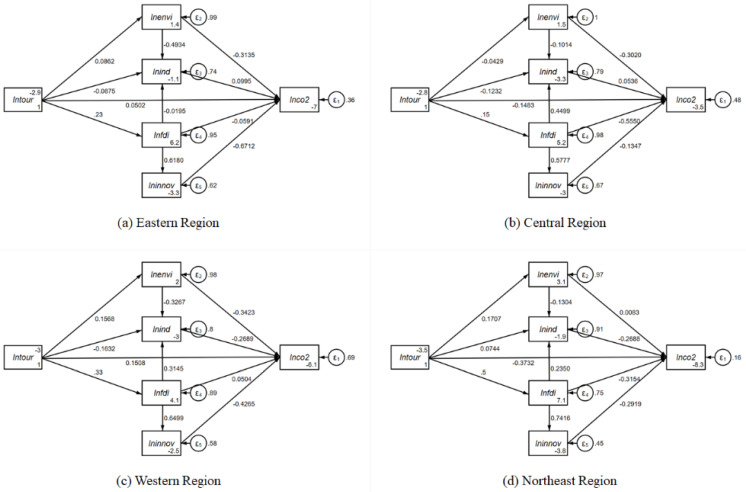
The estimation results of SEM for subsamples.

Furthermore, the heterogeneity of the formation mechanism of the carbon emission reduction effect of tourism economy was investigated. Since the 92 tourism-dependent cities examined are widely distributed across China, the total sample was divided into four subsamples, i.e., eastern, central, western, and northeastern. Then, SEM was estimated based on the perspective of spatial heterogeneity. The effect decomposition results of each region are reported as shown in Figure 7 and Table 5.

The results are as follows: (1) As shown in Table 5, the tourism economy of eastern, central, and northeastern tourism-dependent cities has a significant inhibiting effect on the destination’s carbon emission intensity. The carbon emission reduction effect exists, which has the highest intensity (−0.4289) in the northeast, followed by central (−0.1610) and east (−0.0610). However, tourism economy has no carbon emission reduction effect on western cities. (2) In terms of direct and indirect effects, tourism economy can promote carbon emission reduction in destinations both directly and indirectly in central and northeastern cities, while the carbon emission reduction effect of the tourism economy of eastern cities mainly depends on indirect effects. It was also observed that the tourism economy of western cities has an indirect restrained effect on carbon emission intensity, but it has a direct aggravated effect on the carbon emission intensity of destinations. (3) The difference in the formation mechanism of the carbon emission reduction effect of tourism economy in tourism-dependent cities is shown in Table 6.

## 6. Discussion and Conclusions

### 6.1. Discussion

#### 6.1.1. The Idea of the Carbon Emission Reduction Effect of Tourism Economy

Tourism activities have been confirmed to generate large amounts of greenhouse gases, which has shown that the traditional label of the tourism industry as a green and smoke-free industry has been questioned. Compared with that of other industries, the impact of tourism economy on destinations is more comprehensive and systematic. Therefore, not only should we explore the environmental externalities of tourism from the perspective of its own carbon emissions, but also from a macro perspective, that is, from the impact of tourism on the overall carbon emissions of the destination. Hence, this study confirms that in the context of China’s practices, tourism economy has a mitigation effect on the overall carbon emissions of a destination, especially for tourism-dependent cities. In general, this is attributed to the fact that tourism development needs to be based on a good ecological environment of destinations. This means that the destination’s government must impose strict and effective environmental regulations. The deindustrialization effect of tourism economy has also profoundly affected the industrial structure of destinations. A large number of industries with high pollution and high energy consumption have been excluded from the industrial system, thus reducing carbon emission intensity. In addition, tourism economy increases the opening-up of destinations, and the impact of FDI on innovation capacity has promoted the overall technological progress of destinations, which is ultimately conducive to a reduction in carbon emission intensity.

#### 6.1.2. The Theoretical and Practical Contributions

The main theoretical contributions of this study are as follows: (1) This research enriches the empirical results of tourism economy’s carbon emission reduction effect and, to a certain extent, improves the current situation of insufficient empirical research on this topic. (2) This study opens the black box of the formation mechanism of the carbon emission reduction effect of tourism economy and further confirms the positive environmental externalities of tourism economy in terms of carbon emission reduction. (3) Existing studies on the relationship between tourism and carbon emissions were primarily focused on the national or provincial levels. We conducted empirical research at the prefecture-city scale, which forms a certain complement to this field. Moreover, there are some potential practical contributions of this study. (1) Governments’ understanding of the positive externalities of tourism economy can be strengthened. This is of great significance for encouraging the government to develop tourism as a strategic pillar industry of the national economy that the destination can rely on for a long time. (2) This study is conducive to building a policy system for tourism to promote green and low-carbon transformation, which is important for tourism-dependent cities to explore green and low-carbon transformation pathways against the background of the goals of carbon peak and carbon neutrality.

#### 6.1.3. Limitations and Future Directions

In this manuscript, the study of the carbon emission reduction effect of tourism economy remains at a preliminary stage. There are certain limitations to this research. (1) This research is a preliminary discussion on the formation mechanism of the carbon emission reduction effect of tourism economy, so it needs to be advanced through multiscale comprehensive investigations in future. (2) This study analyzed the carbon emission reduction effect of tourism economy only descriptively through SEM. The limitation of SEM motivates future research to investigate the carbon emission reduction effect through the analysis perspectives of nonlinearity, spatiality, and multidimensional heterogeneity. (3) We have discussed the impact of tourism economy on carbon emissions but ignored the possible heterogeneous effects of different types of tourism on industrial development, innovation capacity, openness, and environmental regulation. An exploration of this heterogeneity is also needed through the development of microscopic survey data in future.

### 6.2. Concluding Remarks

The core aim of this research was to adopt the SEM approach and employ the panel data of 92 tourism-dependent cities to investigate the carbon emission reduction effect of tourism economy and its formation mechanism. Specifically, we examined the existence of a carbon emission reduction effect of tourism economy in China in the first step. As a second step, the transmission mechanism and spatial heterogeneity of the carbon emission reduction effect of tourism economy were discussed. The main conclusions of the research are as follows.

(1) The total carbon emission of tourism-dependent cities in China increased year by year during the study period, while carbon emission intensity decreased. Tourism-dependent cities still face great challenges around carbon emission reduction. Both total carbon emission and carbon emission intensity show a north–south spatial differentiation pattern;

(2) An increase in ln*tour* by 1% can significantly reduce ln*CI* by 0.054% for the whole sample, indicating that tourism economy has a significant carbon emission reduction effect. However, the direct effect of tourism economy on carbon emission intensity is significantly positive;

(3) The carbon emission reduction effect of tourism economy presents multiple pathway characteristics. There are single intermediary pathways such as ln*tour* → ln*envi* → ln*CI* and ln*tour* → ln*open* → ln*CI* and dual intermediary pathways such as ln*tour* → ln*open* → ln*ind* → ln*CI* or ln*tour* → ln*open* → ln*innov* → ln*CI*. The indirect effect formed by the combination of the above pathways can reverse the direct effect of tourism economy on increasing carbon emission intensity.

(4) The formation mechanism of a carbon emission reduction effect of tourism economy has a significant spatial difference in the subsamples (eastern, central, western, and northeastern).

In China, the tourism-dependent development model can provide support for promoting carbon emission reduction, which has been relatively mature. Tourism economy has profoundly affected and reconstructed destination systems, such as economic and social systems and ecosystems of tourism-dependent cities, so that its indirect effect on regional carbon emission intensity contributes to the carbon emission reduction effect. Additionally, tourism economy has a negative direct impact on regional carbon emission intensity, indicating that its responsibility in greenhouse gas emission reduction cannot be ignored. Green, low-carbon, and sustainable are still the realistic demand and mission of high-quality development of tourism economy in this era. According to the results of heterogeneity analysis, local governments should formulate spatial-targeted policies to strengthen the carbon emission reduction effect of tourism economy by focusing on spatial differences in the formation mechanism of the carbon emission reduction effect in tourism economy.

## Figures and Tables

**Table 1:** Variable definitions and references.

Variable	Definition	Calculation Method	Unit	References
Carbon Emission Intensity	Carbon emissions per unit GDP	Ratio method	%	[61,62,63]
Tourism Economy	The proportion of total tourism revenue in GDP	Ratio method	%	[64]
Industrial Development	The proportion of industrial added value in GDP	Ratio method	%	[65]
Innovation Capacity	The innovation index of prefecture-level cities	Get directly	index	[66]
Opening-up	The capital stock of FDI per capita	Perpetual inventory method	CNY/people	[67]
Environmental Regulation	Environmental regulation composite index	Composite index	index	[68]

**Table 2:** Summary statistics and data sources.

Variable	Observation	Mean	SD	Minimum	Median	Maximum	Variable Sources
ln*CI*	1104	2.689	0.798	0.544	2.661	4.511	Self-calculated
ln*tour*	1104	−2.535	0.866	−6.383	−2.472	0.021	China Statistical Yearbook (2006–2017)
ln*envi*	1104	2.380	1.680	−2.940	2.078	11.513	
ln*ind*	1104	−0.983	0.689	−4.074	−0.937	2.343	
ln*open*	1104	6.671	1.869	−0.250	6.852	10.776	
ln*innov*	1104	−0.395	1.731	−6.908	−0.554	5.390	Report on Innovation in Chinese cities and Industries

**Table 3:** Maximum likelihood estimation results of SEM.

		Standardized Coefficients	Standard Error	Z-Value	*p*-Value	[95% Conf. Interval]	
Structural							
ln*CI*	←						
	ln*envi*	−0.1112	0.0095	−11.6700	0.0000	−0.1299	−0.0926
	ln*ind*	−0.0989	0.0245	−4.0400	0.0000	−0.1469	−0.0509
	ln*open*	−0.0286	0.0109	−2.6400	0.0080	−0.0499	−0.0074
	ln*innov*	−0.1377	0.0117	−11.7700	0.0000	−0.1607	−0.1148
	ln*tour*	0.0313	0.0184	1.7000	0.0890	−0.0048	0.0673
	_cons	−3.4647	0.1043	−33.2000	0.0000	−3.6693	−3.2602
ln*envi*	←						
	ln*tour*	0.1313	0.0582	2.2500	0.0240	0.0172	0.2455
	_cons	2.7126	0.1560	17.3900	0.0000	2.4069	3.0184
ln*ind*	←						
	ln*envi*	−0.1231	0.0112	−11.0200	0.0000	−0.1450	−0.1012
	ln*open*	0.1119	0.0105	10.6300	0.0000	0.0913	0.1325
	ln*tour*	−0.0827	0.0227	−3.6400	0.0000	−0.1272	−0.0382
	_cons	−1.6458	0.1119	−14.7100	0.0000	−1.8651	−1.4266
ln*open*	←						
	ln*tour*	0.6617	0.0618	10.7000	0.0000	0.5405	0.7829
	_cons	8.3484	0.1656	50.4100	0.0000	8.0238	8.6730
ln*innov*	←						
	ln*open*	0.5987	0.0213	28.1500	0.0000	0.5570	0.6404
	_cons	−4.3886	0.1474	−29.7800	0.0000	−4.6774	−4.0998
Variance							
var(e. ln*CI*)		0.2467	0.0105			0.2270	0.2682
var(e. ln*envi*)		2.8062	0.1194			2.5816	3.0504
var(e. ln*ind*)		0.3831	0.0163			0.3524	0.4164
var(e. ln*open*)		3.1625	0.1346			2.9093	3.4376
var(e. ln*innov*)		1.7433	0.0742			1.6037	1.8949

Note: Number of observations: 1104. Log likelihood: −9449.4526. LR test of model vs. saturated: chi2(4) = 84.00, Prob > chi2 = 0.0000. Goodness of fit: RMSEA: 0.135. AIC: 18,940.905. BIC: 19046.046. CFI: 0.942. SRMR: 0.068.

**Table 4:** Direct and indirect effects of tourism economy on carbon emission intensity.

		Direct Effect	Indirect Effect	Total Effect
ln*CI*				
	ln*envi*	−0.1112 ***	0.0122 ***	−0.0991 ***
		(0.0095)	(0.0032)	(0.0092)
	ln*ind*	−0.0989 ***	(no pathway)	−0.0989 ***
		(0.0245)		(0.0245)
	ln*open*	−0.0286 ***	−0.0935 ***	−0.1222 ***
		(0.0109)	(0.0077)	(0.0090)
	ln*innov*	−0.1377 ***	(no pathway)	−0.1377 ***
		(0.0117)		(0.0117)
	ln*tour*	0.0313 *	−0.0857 ***	−0.0544 ***
ln*envi*		(0.0184)	(0.0117)	(0.0199)
	ln*tour*	0.1313 **	(no pathway)	0.1313 **
		(0.0582)		(0.0582)
ln*ind*				
	ln*envi*	−0.1231 ***	(no pathway)	−0.1231 ***
		(0.0112)		(0.0112)
	ln*open*	0.1119 ***	(no pathway)	0.1119 ***
		(0.0105)		(0.0105)
	ln*tour*	−0.0827 ***	0.0579 ***	−0.0248
		(0.0227)	(0.0123)	(0.0237)
ln*open*				
	ln*tour*	0.6617 ***	(no pathway)	0.6617 ***
		(0.0618)		(0.0618)
ln*innov*				
	ln*open*	0.5987 ***	(no pathway)	0.5987 ***
		(0.0213)		(0.0213)
	ln*tour*	(no pathway)	0.3961 ***	0.3961 ***
			(0.0396)	(0.0396)

Note: ***, **, * represent 1%, 5%, and 10% significance level, respectively.

**Table 5:** Analysis of spatial heterogeneity.

		East	Central	West	Northwest
Direct Effect					
ln*CI*					
	ln*envi*	−0.0696 ***	−0.1517 ***	−0.1252 ***	0.004
	ln*ind*	0.0803 **	0.0441	−0.2976 ***	−0.1329 ***
	ln*open*	−0.0212	−0.2210 ***	0.0178	−0.0948 ***
	ln*innov*	−0.1946 ***	−0.0540 **	−0.1459 ***	−0.0969 ***
	ln*tour*	0.0303	−0.1030 ***	0.1059 ***	−0.2336 ***
ln*envi*					
	ln*tour*	0.2346	−0.0593	0.3010 ***	0.2206 *
ln*ind*					
	ln*envi*	−0.1358 ***	−0.0619 **	−0.1080 ***	−0.1278
	ln*open*	−0.0087	0.2179 ***	0.1005 ***	0.1429 **
	ln*tour*	−0.0656	−0.1041 **	−0.1036 ***	0.0943
ln*open*					
	ln*tour*	0.3937 ***	0.2580 ***	0.6540 ***	1.0477 ***
ln*innov*					
	ln*open*	0.7639 ***	0.5737 ***	0.6721 ***	0.6714 ***
Indirect Effect					
ln*CI*					
	ln*envi*	−0.0109 **	−0.0027	0.0321 ***	0.0170
	ln*open*	−0.1493 ***	−0.0214 *	−0.1280 ***	−0.0840 ***
	ln*tour*	−0.0913 ***	−0.0580 **	−0.0692 ***	−0.1953 ***
ln*ind*					
	ln*tour*	−0.0353	0.0599 ***	0.0333 *	0.1215
ln*innov*					
	ln*tour*	0.3007 ***	0.1480 ***	0.4395 ***	0.7034 ***
Total Effect					
ln*CI*					
	ln*envi*	−0.0805 ***	−0.1544 ***	−0.0931 ***	0.021
	ln*ind*	0.0803 **	0.0441	−0.2976 ***	−0.1329 ***
	ln*open*	−0.1705 ***	−0.2425 ***	−0.1102 ***	−0.1788 ***
	ln*innov*	−0.1946 ***	−0.0540 **	−0.1459 ***	−0.0969 ***
	ln*tour*	−0.0610 *	−0.1610 ***	0.0367	−0.4289 ***
ln*envi*					
	ln*tour*	0.2346	−0.0593	0.3010 ***	0.2206 *
ln*ind*					
	ln*envi*	−0.1358 ***	−0.0619 **	−0.1080 ***	−0.1278
	ln*open*	−0.0087	0.2179 ***	0.1005 ***	0.1429 ***
	ln*tour*	−0.1008 **	−0.0442	0.0703 **	0.2158 *
ln*open*					
	ln*tour*	0.3937 ***	0.2580 ***	0.6540 ***	1.0477 ***
ln*innov*					
	ln*open*	0.7639 ***	0.5737 ***	0.6721 ***	0.6714 ***
	ln*tour*	0.3007 ***	0.1480 ***	0.4395 ***	0.7034 ***

Note: ***, **, * represent 1%, 5%, and 10% significance level, respectively.

**Table 6:** Spatial heterogeneity of carbon emission reduction effects in tourism economy.

Pathways		East	Central	West	Northwest
Direct Effect	ln*tour* → ln*CI*	◯	-	++	-
Total Indirect Effect	Indirect Effects of Aggregation of Pathways	++	++	++	++
Single Intermediary pathways	ln*tour* → ln*envi* → ln*CI*	+	◯	++	◯
	ln*tour* → ln*ind* → ln*CI*	++	+	++	++
	ln*tour* → ln*open* → ln*CI*	◯	++	◯	++
Dual Intermediary pathways	ln*tour* → ln*envi* → ln*ind* → ln*CI*	+	◯	-	-
	ln*tour* → ln*open* → ln*ind* → ln*CI*	◯	◯	-	-
	ln*tour* → ln*open* → ln*innov* → ln*CI*	++	++	++	++

Note: ++ indicates that the pathway direction conforms to the hypothesis and is significant. + indicates that the pathway direction conforms to the hypothesis but is not significant. ◯ indicates that the pathway direction is inconsistent with the hypothesis and is not significant. - indicates that the pathway direction is inconsistent with the hypothesis and significant. Significance is defined at the level of 10%.

## Data Availability

Not applicable.
